# Differential hippocampal protein expression between normal mice and mice with the perioperative neurocognitive disorder: a proteomic analysis

**DOI:** 10.1186/s40001-021-00599-3

**Published:** 2021-11-03

**Authors:** Chuan Li, Jingzhu Li, He Tao, Jinghua Shan, Fanghao Liu, Xiyuan Deng, Yanan Lin, Xu Lin, Li Fu, Bin Wang, Yanlin Bi

**Affiliations:** 1 Department of Anesthesiology, Qingdao Municipal Hospital Affiliated to Qingdao University, 5 Donghai Middle Road, Qingdao, Shandong 266071 People’s Republic of China; 2 Department of Anesthesiology, Weifang Medical University, Weifang, Shandong China; 3 Department of Anesthesiology, Dalian Medical University, Dalian, Liaoning China

**Keywords:** Cognition disorders, Hippocampus, Proteomics, Liquid chromatography–mass spectrometry, Gene ontology, Kyoto Encyclopedia of Genes and Genomes

## Abstract

**Objectives:**

To compare differential expression protein in hippocampal tissues from mice of perioperative neurocognitive disorder (PND) and normal control mice and to explore the possible mechanism of PND.

**Methods:**

Mice were randomly divided into a PND group (*n* = 9) and a control group (*n* = 9).The mice in the PND group were treated with open tibial fracture with intramedullary fixation under isoflurane anesthesia, while the mice in the control group received pure oxygen without surgery. The cognitive functions of the two groups were examined using Morris water maze experiment, Open field test and Fear conditioning test. The protein expression of the hippocampus of mice was analyzed by high-performance liquid chromatography–mass spectrometry (HPLC–MS/MS). Gene ontology (GO) and Kyoto Encyclopedia of Genes and Genomes (KEGG) pathway enrichment analyses were performed to explore the principal functions of dysregulated proteins.

**Results:**

A total of 21 proteins were differentially expressed between PND and control mice on days 1, 3, and 7 after the operation. These proteins were involved in many pathological processes, such as neuroinflammatory responses, mitochondrial oxidative stress, impaired synaptic plasticity, and neuronal cell apoptosis. Also, the dysregulated proteins were involved in MAPK, AMPK, and ErbB signaling pathways.

**Conclusion:**

The occurrence of PND could be attributed to multiple mechanisms.

Perioperative neurocognitive disorder (PND) is a common neuropsychological complication in patients during the perioperative period. It affects all aspects of cognitive function, such as learning, memory, attention, and executive function, and is more common in elderly patients [1]. PND increases the risk of dementia and is highly associated with the occurrence and progression of Alzheimer’s disease (AD) [2]. PND often leads to a variety of adverse consequences, such as prolonged hospitalization, reduced quality of life, increased disability, and mortality [3]. However, the specific mechanisms underlying PND remain unclear. These mechanisms include β-amyloid (Aβ) deposition, phosphorylation of tau proteins, central nervous inflammation, oxidative stress, central nervous neurotransmitter homeostasis, and neuronal apoptosis [4–6]. Data-dependent acquisition (DDA) combined with the data-independent acquisition (DIA) is the mainstream technology of proteomics and an effective way to explore the mechanism of disease [7]. The hippocampus plays a major role in learning and memorizing [8, 9]. Some studies have shown that the volume of the hippocampal area in PND patients is reduced [10]. Thus, the present study aimed to explore the putative molecular and biological mechanisms of PND by screening the differentially expressed proteins in the hippocampus of normal and PND mice.

## Materials and methods

### Laboratory animals and groups

C57BL/6 mice used in this study were provided by the Hubei Medical Animal Experimental Center (Hubei, China). The mice were male, aged 15 months, and weighed 30–35 g. The animals were raised at a constant temperature with a 12/12‑h light/dark cycle, 20–25 ℃ and had free access to chow and water. A random number table method was used to divide the animals into two groups: control group (C group, *n* = 9), PND (*P* group, *n* = 9). All cognitive function tests were performed under light conditions.

### Animal model of PND

Isoflurane anesthesia was administered to mice in the PND group to establish a PND model. The PND group was subjected to open tibial fracture with intramedullary fixation under isoflurane anesthesia (induced by 3.0% isoflurane, maintained under 1.5% isoflurane in pure oxygen) while mice in the control group received 40% oxygen for 2 h without surgery. Butorphanol 0.1 mg/kg was injected subcutaneously before skin incision. The general anesthesia time was about 30 min. After local skin preparation, a skin incision of about 1 cm was made at the tibial tuberosity under the knee. The tibia was exposed after separating the muscle and mucosal tissue. The periosteum was peeled about 1 cm along the tibia. The intramedullary needle was inserted into the medullary cavity after the horizontal drilling of the tibial tuberosity to achieve internal fixation. The tibia was transected by a blade at the junction of the middle and lower 1/3 of the tibia. After local debridement, the wound was sutured with 4–0 suture. Subsequently, the mice were placed in a cage with pure oxygen until consciousness recovered, and lidocaine ointment was applied locally with every 8 h in 3 days after operation. Aseptic conditions were maintained during the operation.

### Cognitive function test (CFT)

#### Morris water maze experiment

Water maze was a cylindrical tank with 120 cm diameter consisting of a platform with 10 cm diameter, filled with opaque water obscuring the platform (water 2 cm above the platform height).

*Orientation navigation experiment* The platform was located in the center of a quadrant. On each acquisition day (5 days preoperative until operation), the mice underwent four consecutive tests (60 s each with a 20-min interval) to find the hidden platform. The escape latency was recorded as the time when the mice were on the platform. If the platform was not found, the animals were guided to the platform, removed after 60 s and placed in a holding cage.

*Space exploration experiment* The platform was removed 1 day before the operation. The escape latency and % residence time in the target quadrant were recorded. The operation group was subjected to the space exploration experiment on days 1, 3, and 7 after the operation.

#### Open field test (OFT)

OFT was used to evaluate the anxiety and locomotor activity in experimental animals. A mouse was placed directly into the center of the open field (100 cm × 100 cm × 48 cm, length × width × height) in dim light. The animal movements were recorded by an animal tracking system during the 5-min testing sessions. Then, the total distance of motion was calculated. After each test, the open field was wiped with 75% ethanol.

#### Fear conditioning test (FCT)

The purpose of FCT was to investigate the animals’ abilities to learn and memorize associations between unpleasant experiences and environmental implications. Sound is commonly used as a conditioning stimulus (CS), while an aversive stimulus (such as an electric shock to the foot) acts as an unconditional stimulus (US). The two types of stimuli appear in pairs during the test. After CS–US training, in addition to the association between the sound and electric shock, animals were able to link the electric shock and the surrounding environment.

One day prior to the operation, after a 2-min exploration period, mice were given six pairs of sound stimuli (2000 Hz, 70 db, 20 s) and electric shock stimuli (0.7 mA, 2 s). Each electric shock stimulus appeared in the last 2 s of the corresponding sound stimulus and both ended at the same time. An interval of 1 min was maintained between the two pairs of stimuli.

On days 1, 3, and 7 following the operation, the contextual fear memory test was performed. During the test, the mice were placed in a context similar to that of the preoperative training for a 5-min observation period without the stimulation of sound and electric shock. The percentage of freezing time was recorded. At 2 h after the completion of the contextual test, the auditory fear memory test was carried out, for which, the mice were placed in a context different from that during the preoperative training (the interior was changed). After a 5-min exploration period, mice were given six sound stimuli (2000 Hz, 90 db, and 30 s) without an electric shock stimulus. The observation time was 5 min. Then, the percentage of freezing time of the mice in the auditory fear memory test was calculated and five minutes were used as total time.

### Brain tissue collection and treatment

#### Hippocampal tissue collection

Three mice from the PND group with poor cognitive function test results after the operation and three mice selected randomly from control group were killed under anesthesia by decapitation on days 1, 3, and 7 postoperation. The brain tissue was removed, the hippocampus was stripped, and preserved at – 80 ℃ for subsequent proteomic analysis.

#### Hippocampal tissue protein extraction

The tissues were lysed in sodium dodecyl sulfate (SDS)-lysis buffer and homogenized for 10 min before centrifugation at 12,000×g to remove the cell debris. The purified protein extract in the supernatant was precipitated by precooled ethanol and lytic solution. Finally, the protein concentration was estimated by Bradford assay, and the samples were stored at – 80 ℃.

From each sample, 100 μg of total protein sample was processed according to the FASP (filter-aided sample preparation) protocol [11]. Briefly, 4.5 mM DTT was added for reduction of the sample (at 37 ℃ for 1 h), 10 mM IAA for alkylation (room temperature, 30 min), and trypsin for enzymatic hydrolysis (37 ℃, 16 h). The peptide fragments were recovered after enzymatic hydrolysis and desalted on an Oasis HLB column. The peptide segments were lyophilized into powder, estimated by the Bradford assay, and preserved at − 80 ℃.

#### High pH small column fractions

The mixed sample, consisting of 10 μg peptide segments from each sample, was applied to high pH column (pH = 10) for gradient elution (acetonitrile concentration of the eluent was 5%, 7.5%, 10%, 12.5%, 15%, 17.5%, 20%, and 50%, respectively). The ten components after elution were lyophilized into peptide powder for the subsequent mass spectrometry analysis.

### High-performance liquid chromatography–mass spectrometry (HPLC–MS/MS) analysis

#### Establishment of spectrogram database and data acquisition

A total of 10 components were resolubilized in 20 μL of 0.1% formic acid. Of this, 9 μL of each sample was mixed with 1 μL of iRT peptide. Finally, 3 μL of the mixture was subjected to HPLC (Thermo EASY-nLC™ 1200, ThermoFisher Scientific, Massachusetts, USA): the gradient elution was 4–28% mobile phase B (79.9% acetonitrile, 20% water, 0.1% formic acid) over 60 min at a flow rate of 0.3 μL/min. The peptides eluted by reversed-phase column (C18, 15 cm long, internal diameter 50 μm) were identified by Orbitrap Fusion Lumos™ mass spectrometer (spray voltage 2.1 kV, ion transport tube temperature 320 ℃). MS1 full scan with 120,000 resolution (mass range 350–1550 m/z) and automatic gain control (AGC) was 1 × 10^5^. The maximum injection time was 100 ms. MS2 was followed by data-dependent acquisition (30,000 resolution, maximum speed mode, cycle time 3 s) using higher-energy collision dissociation (HCD) at 30% normalized collision energy. The AGC was 5 × 10^4^, and the maximum injection time was 50 ms.

#### DIA data collection

Each peptide fragment sample was solubilized in 0.1% formic acid (concentration 0.5 μg/μL, and 1 μL iRT was added to 9 μL of the sample. A volume of 2 μL of the sample mixture was analyzed by mass spectrometry. The liquid phase method was consistent with the established spectrogram database. The MS1 full scans with 60,000 resolution (mass range 350–1550 m/z), and AGC was 2 × 10^5^. The maximum injection time was 50 ms. MS2 was followed by data-independent acquisition (30,000 resolution, 32 windows opened, mass range 200–2000 m/z) using HCD at 30% normalized collision energy. The AGC was 5 × 10^5^, and the maximum injection time was 70 ms.

### Data processing and statistical analysis

#### Establishment of the spectrogram library

Ten sets of data were collected by DDA through Proteome Discoverer (version 2.1, Thermo Fisher Scientific, Massachusetts, USA): fully tryptic peptides, up to two missed cleavages allowed. Fixed modifications were carbamidomethylation on cysteine residues. The mass deviation of the parent ion was 10 ppm, and the sub-ion mass deviation was 0.05 Da. The exported pdResult results and the original raw file were imported into the Spectronaut Pulsar software (Biognosys Co, Schlieren, Switzerland). The initial false discovery rate (FDR) for protein identification was set to 1%.

#### DIA data processing

The DIA data were analyzed using Spectronaut Pulsar software. A previously established spectrum library was loaded. Swiss_Prot mouse database was selected as the background library.

### Statistical analysis

SPSS 17.0 (IBM Corp., Armonk, NY, USA) was used for statistical analyses. The measurement data of normal distribution are expressed as mean ± standard deviation. *T*-test was used to screen out markedly altered proteins. The least significant difference (LSD-t) was used to compare between the groups. *P*<0.05 indicated statistical significance.

### Mapping

The results of the DIA were plotted as heatmaps in R, and volcano plots were constructed using GraphPad Prism 8.0 (GraphPad Software Inc., San Diego, CA, USA) to screen out differential expression proteins (fold-change > 1.20 or <0.83, *P *< 0.05) and the corresponding genes encoding the proteins. The common differentially expressed proteins were selected on days 1, 3, and 7 day after the operation using *R*.

### GO analysis and KEGG pathway analysis

The differential expression proteins were introduced into the Database for Annotation, Visualization, and Integrated Discovery (DAVID) Bioinformatics Resources (https://david.ncifcrf.gov/) for GO and KEGG analysis. The data were visualized by *R*, and the *P*-values of KEGG were corrected and ordered by *Q*-values. *Q *< 0.5 indicated statistical significance.

## Results

### Construction of PND mouse model

**Fig. 1:**
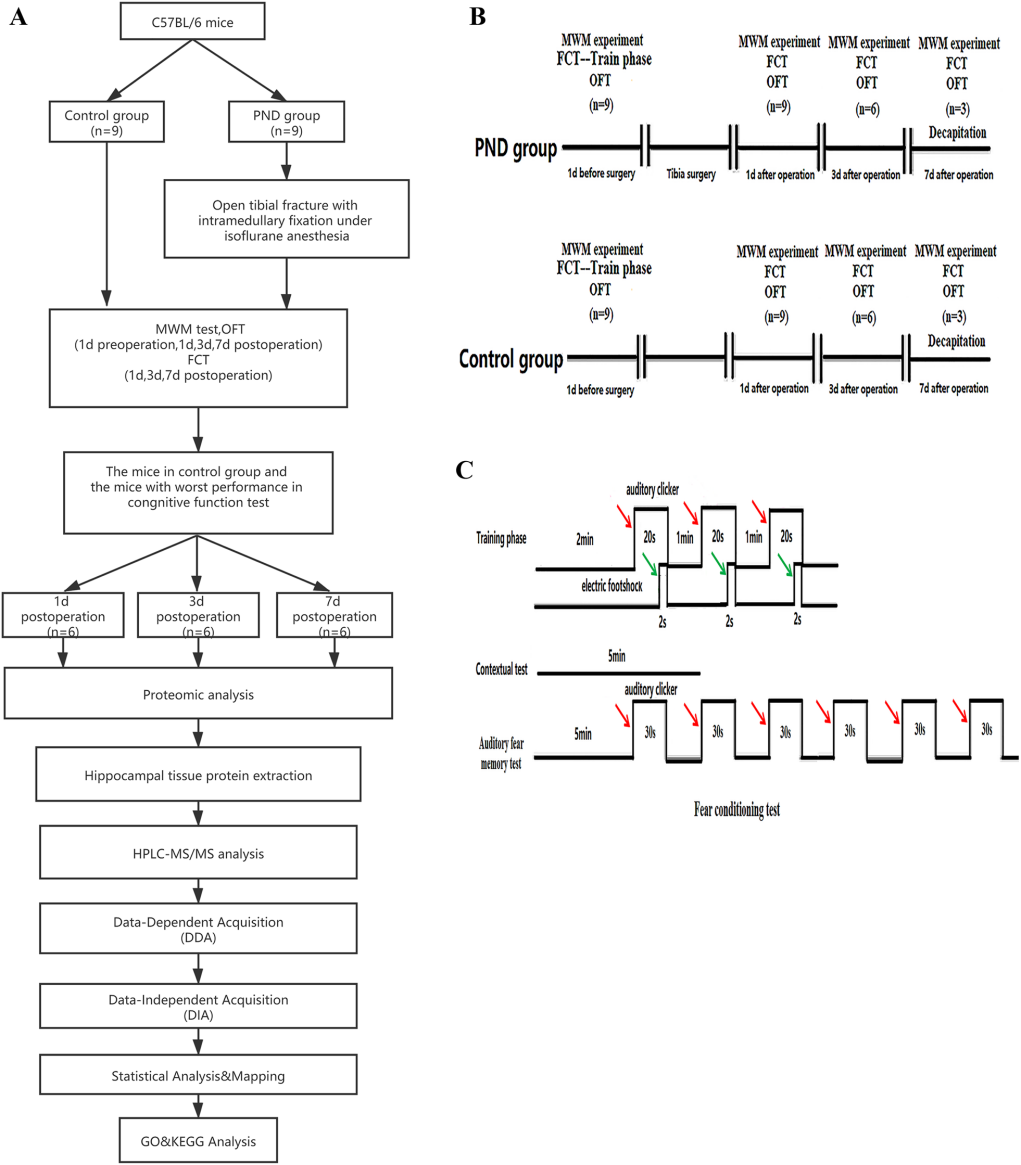
**A** Flowchart detailing the experimental groups and steps performed. PND, perioperative neurocognitive disorder. MWM experiment: Morris water maze experiment. *FCT* fear conditioning test, *OFT* open field test. **B** Animal experimental work flow: the animal experiment was performed according to the figure. MWM experiment: Morris water maze experiment. *FCT* fear conditioning test. *OFT* open field test. **C** Fear conditioning test workflow: red arrows indicate sound stimuli and green arrows indicate electric shock stimuli. The two groups were subjected to behavioral training 1 d prior to surgery (training phase) and behavioral tests were performed on the 1st, 3rd, 7th, day post-surgery (contextual and cued tests)

**Fig. 2:**
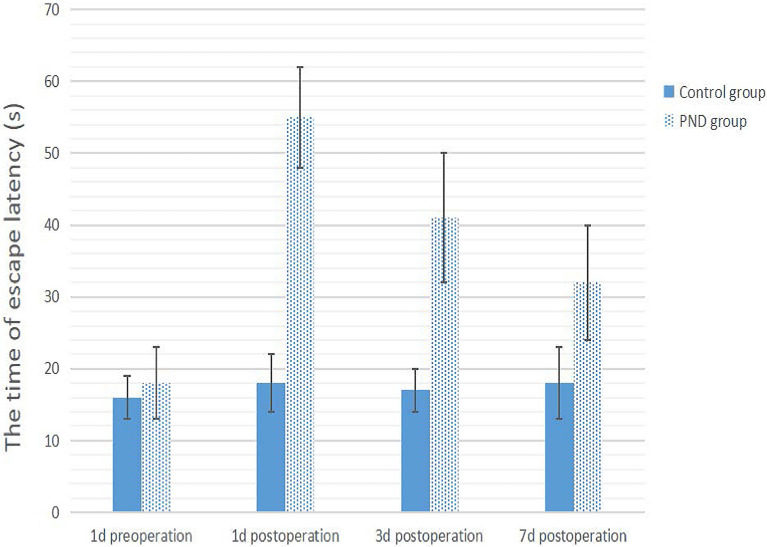
The time of escape latency of control group vs PND group and preoperation vs postoperation. Compared to the preoperation, escape latency of PND group was prolonged postoperation (*P* < 0.05). Escape latency of PND group was increased compared to control group (*P* < 0.05)

**Fig. 3:**
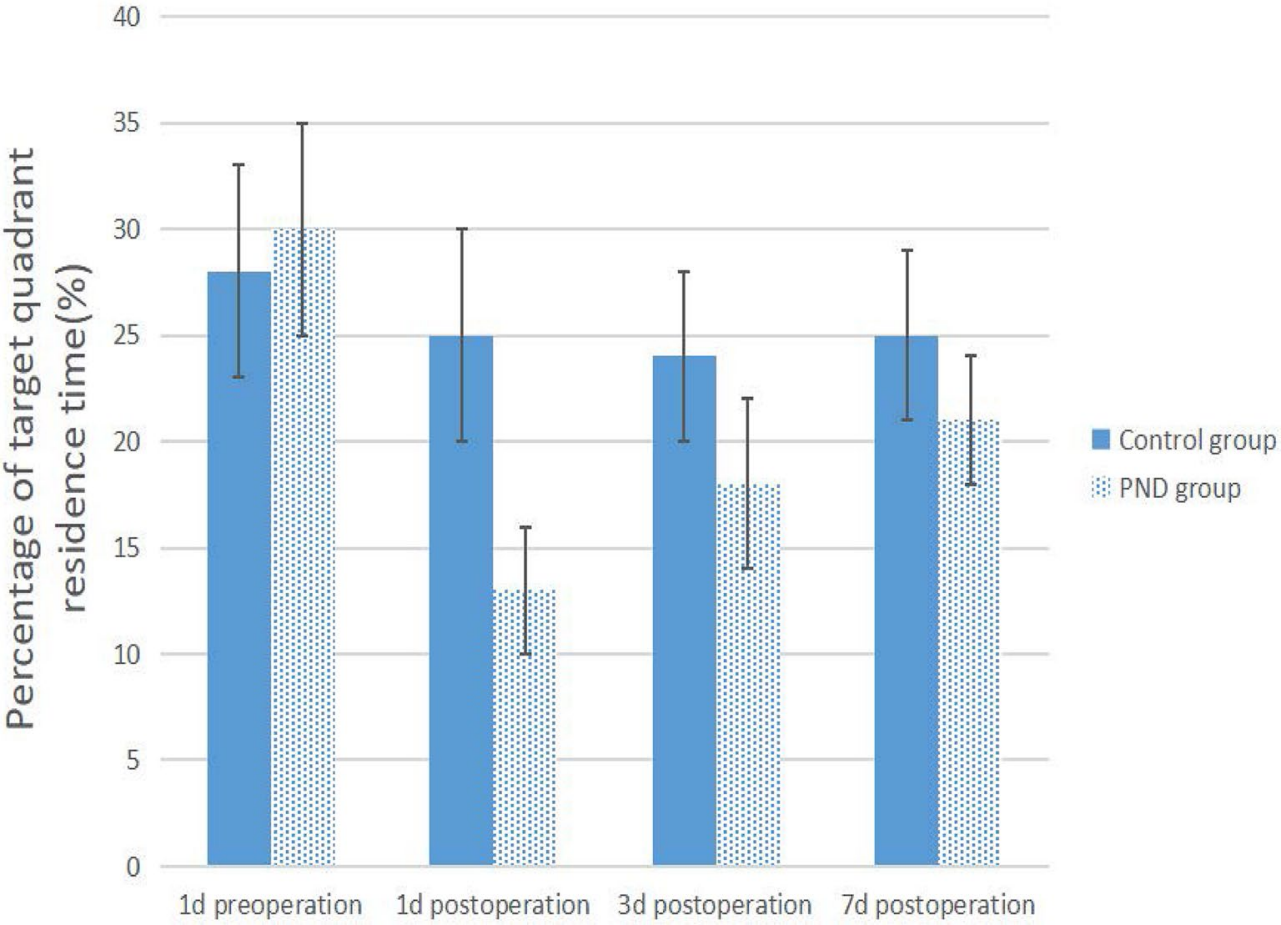
The percentage of target quadrant residence time of PND group and control group. Percentage of target quadrant residence time of PND group was decreased vs preoperation vs control group at first, third, 7th day after operation (*P* < 0.05)

**Fig. 4:**
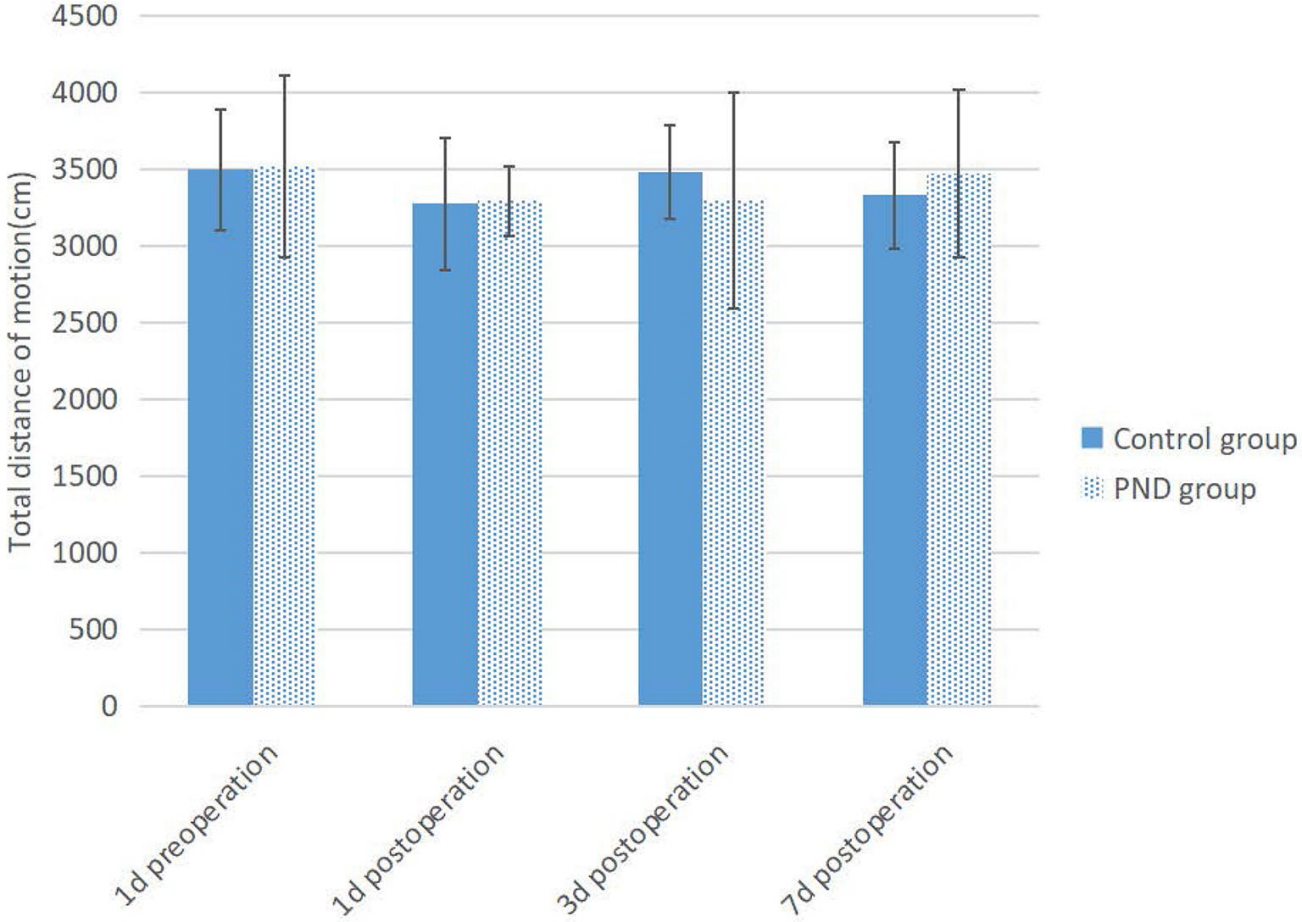
Total distance of motion of PND group and control group. There was no significant difference in total distance between PND group and control group (*P* < 0.05)

**Fig. 5:**
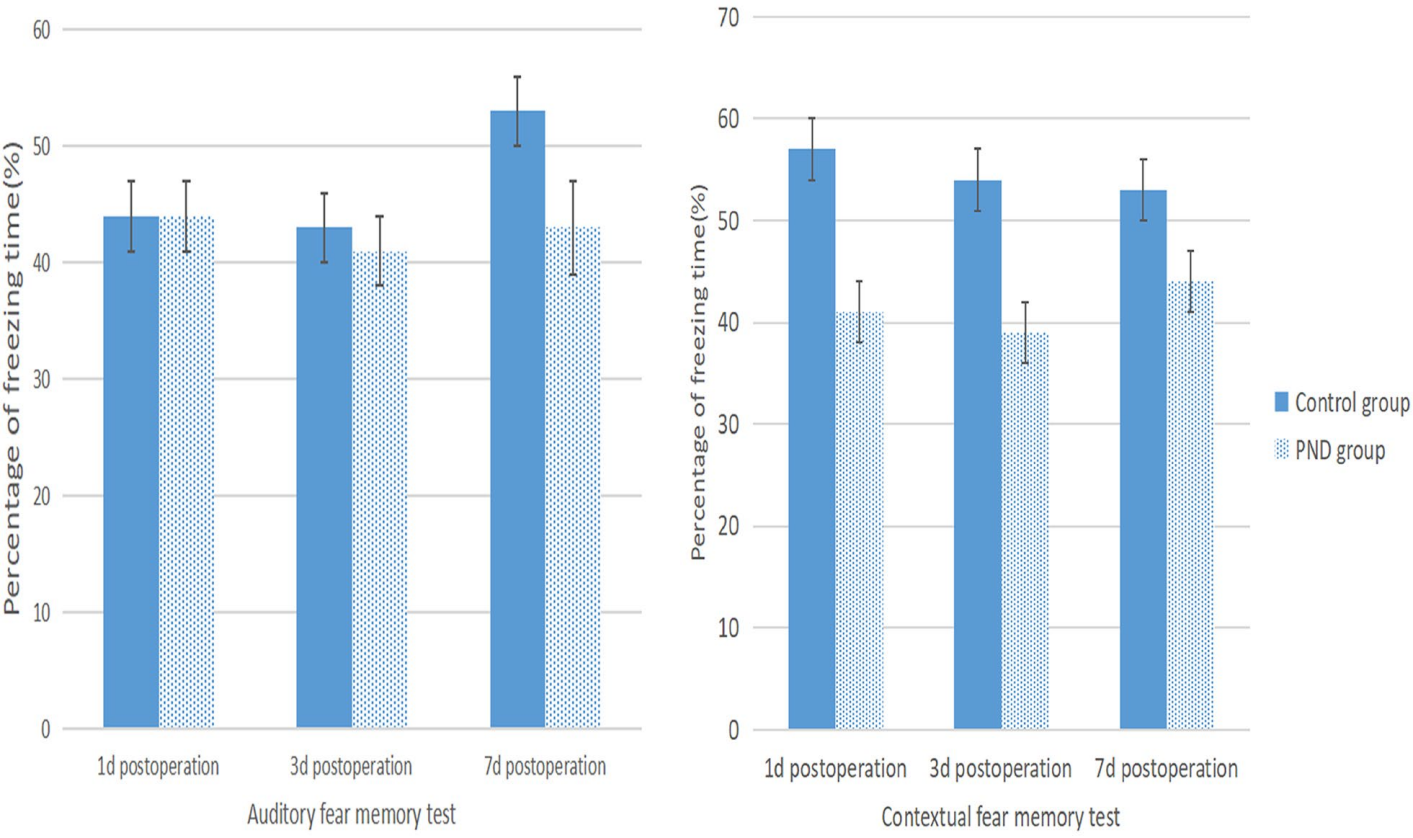
The percentage of freezing time of fear memory tests. The percentage of freezing time of contextual fear memory tests declined in PND group vs control group (*P* < 0.05) and there was no significant difference in the percentage of freezing time of auditory fear memory tests between two groups (*P* > 0.05)

The PND model construction flowchart is shown in Fig. 1. In the current study, Morris water maze test, CFT, and OFT were performed on mice as the basis for the success of PND modeling. Compared to preoperation, the escape latency of the PND group was prolonged (Fig. 2, *P* = 0.0016, *P* = 0.0082, *P* = 0.0098 on 1d, 3d, 7d postoperation, respectively), and the percentage of target quadrant residence time was decreased on days 1, 3, and 7 after the operation (Fig. 3, *P* = 0.0149, *P* = 0.0348, *P* = 0.0489 on 1d, 3d, 7d postoperation, respectively). No significant difference was detected in the total distance of motion (Fig. 4, 1d postoperation *P* = 0.7862, 3d postoperation *P* = 0.8512, 7d postoperation *P* = 0.7859). The escape latency of the PND group was increased (Fig. 2, 1d postoperation *P* = 0.0018, 3d postoperation *P* = 0.0092, 7d postoperation *P* = 0.0197), and the percentage of target quadrant residence time was decreased compared to the control group (Fig. 3, 1d postoperation *P* = 0.0158, 3d postoperation *P* = 0.0384, 7d postoperation *P* = 0.0497) on days 1, 3, and 7 after the operation. However, no significant difference was detected in the total distance between PND and control groups (Fig. 4, 1d postoperation *P* = 0.8231, 3d postoperation *P* = 0.7872,7d postoperation *P* = 0.8566). Compared to the C group, the percentage of freezing time of fear memory experiment decreased (Fig. 5, 1d postoperation *P* = 0.0017, 3d postoperation *P* = 0.0028, 7d postoperation *P* = 0.0053) in the PND group, while no difference was noted in the percentage of freezing time of auditory fear memory test (Fig. 5, 1d postoperation *P* = 0.2080, 3d postoperation *P* = 0.3739, 7d postoperation *P* = 0.9217).

### Access to differential proteins

**Fig. 6:**
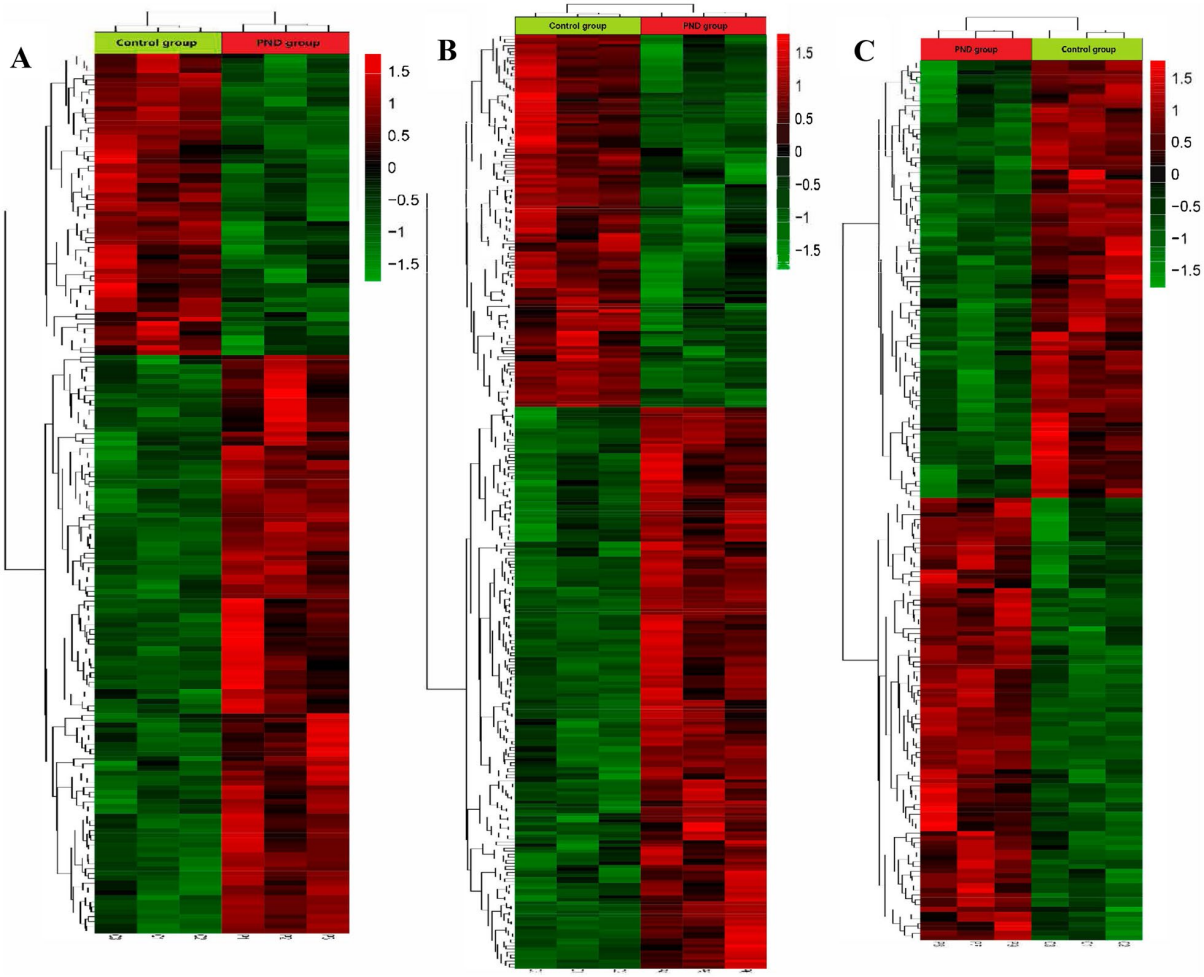
Heatmap showing protein levels at 1d, 3d and 7d postoperation. Screening criteria were: FC > 1.20 OR FC < 0.05 for **A** 1d postoperation PND group vs Control group and for **B** 3d postoperation PND group vs Control group and for **C**. 7d postoperation PND group vs Control group. Expression values are depicted in line with the color scale; intensity increases from green to red. Each column represents one sample, and each row indicates a protein

**Fig. 7:**
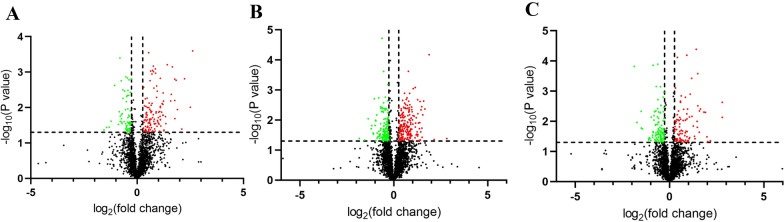
Volcano plots reflect number, significance, and reliability of differentially expressed. **A** 1d postoperation PND group vs Control group and **B** 3d postoperation PND group vs Control group, and **C** 7d postoperation PND group vs Control group. The abscissa is log2 (fold-change) and the ordinate is − log10 (*P* value). Red dots are upregulated genes, green dots are downregulated genes, and black dots are genes that were the same between the two groups

The differentially expressed proteins (fold-change > 1.20 or < 0.83, *P* < 0.05) were screened at each time point. Heat maps (Fig. 6) and volcano plots (Fig. 7) are illustrated. These results displayed 21 commonly differential expression proteins at the three time points after the operation. Among these, 13 proteins were upregulated, and 9 proteins were downregulated (Table 1).

**Table 1:** Details of differentially expressed proteins

Fold-change (1d postoperation, PND vs Control)	Fold-change (3d postoperation, PND vs Control)	Fold-change (7d postoperation, PND vs Control)	Protein function
4.04	5.29	6.93	Cellular potassium ion homeostasis
3.47	2.63	3.11	Heme binding
2.38	3.14	3.73	Positive regulation of MAP kinase activity Negative regulation of protein catabolic process
1.99	2.24	2.83	Mitochondrial outer membrane
2.36	2.73	2.40	Positive regulation of apoptotic process
2.59	2.29	2.01	Structural constituent of ribosome
2.23	1.76	1.89	Cytoplasm
2.01	2.22	1.99	SNARE binding
1.79	2.01	2.08	Pyruvate metabolism
1.87	1.54	1.49	Structural constituent of ribosome
1.98	1.88	1.44	1-Phosphatidylinositol binding
1.36	1.76	1.68	Biological process
0.66	0.70	0.74	Proteasome-mediated ubiquitin-dependent protein catabolic process
0.77	0.73	0.68	U1 snRNP binding
0.72	0.68	0.67	Structural constituent of ribosome
0.63	0.68	0.79	Protein binding
0.59	0.80	0.73	Negative regulation of dopamine metabolic process
0.61	0.61	0.43	Structural constituent of ribosome
0.54	0.52	0.27	Nucleus
0.54	0.52	0.27	Nucleus
0.57	0.58	0.66	Biological process

The commonly differential expression proteins at 1d, 3d, 7d postoperation (fold-change > 1.20 or fold-change < 0.83) were screened out. Fold-change > 1.20 means upregulation, and < 0.05 means downregulation (*P* < 0.05)

### GO analysis

**Fig. 8:**
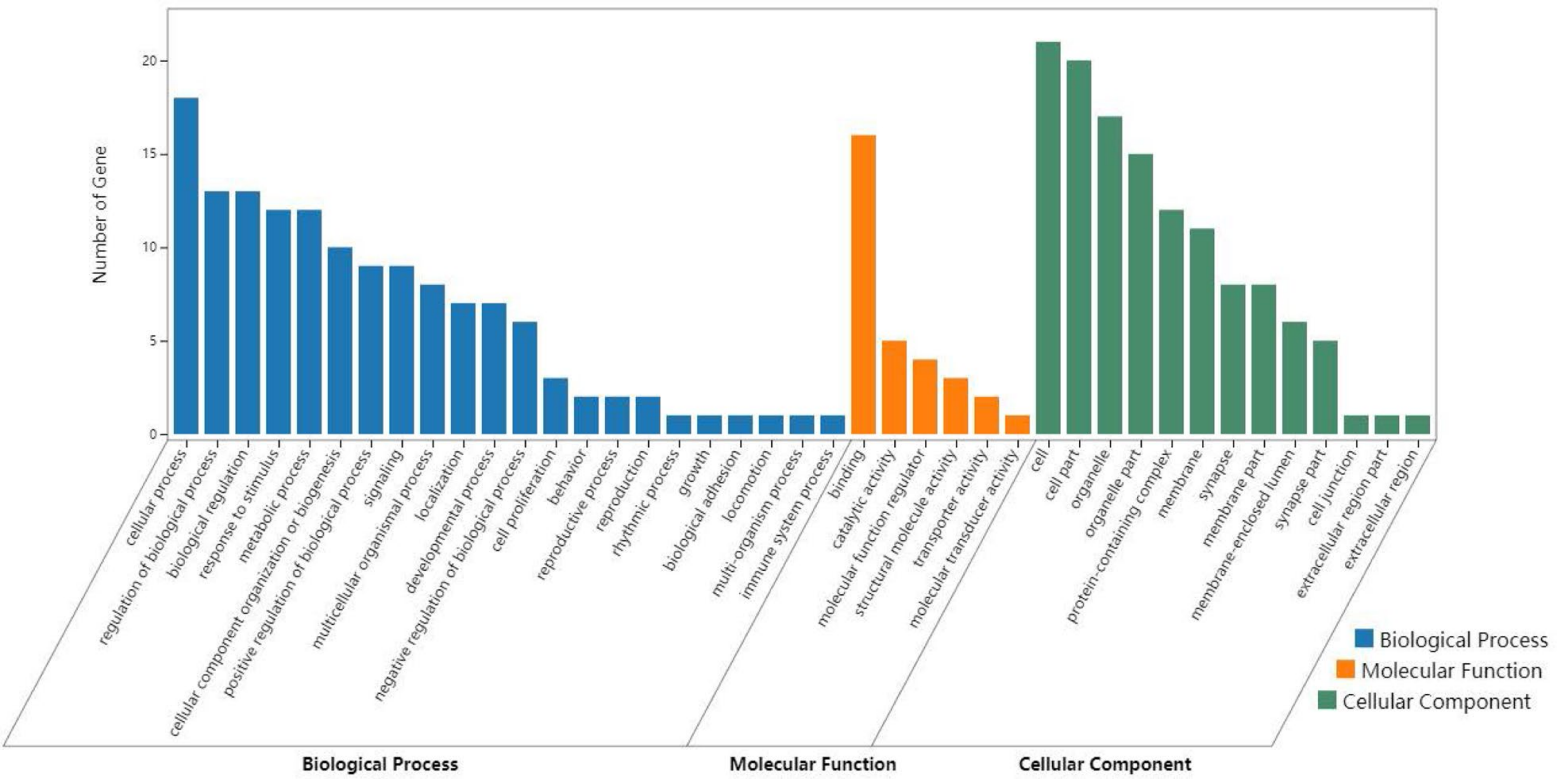
The GO analysis of differentially expressed protein. The abscissa of the chart is the classification of the GO database. The left ordinate is the number of genes. Blue bars are biological processes, green are cellular components, and orange are molecular functions

DAVID was used for GO enrichment analysis of differentially expressed proteins. We found that the differentially expressed proteins are involved in the stimulation and metabolism of cells, which constitute organelles, synapses, and other structures, and are related to molecular functions, such as binding and transport (Fig. 8).

### KEGG pathway analysis

**Fig. 9:**
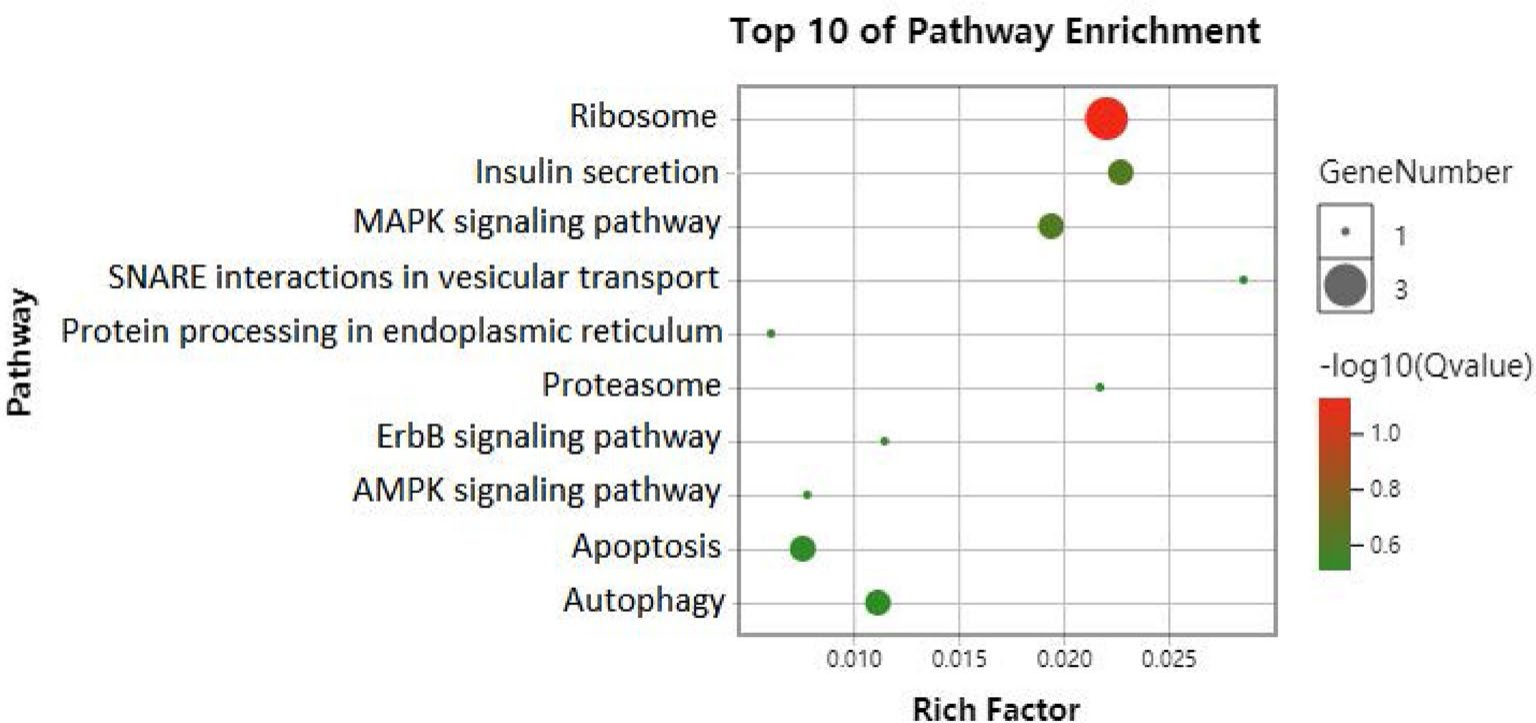
KEGG analysis of differential proteins is shown by bubble plot. Compare the results obtained in the previous step with the background genes, and identify significant enrichment pathways. Statistical testing was performed using hypergeometric distribution. The abscissa is Rich Factor. The higher the Rich Factor, the greater the enrichment. *Q* value is *p* value through multiple test. The redder the points, the bigger the − Lg*Q*, indicating that enrichment of differentially expressed proteins in a given pathway was significant

KEGG pathway analysis of significantly differential proteins was applied by DAVID (Fig. 9). This analysis (selecting 10 pathways with the highest enrichment scores) showed that the differentially expressed proteins were mainly involved in MAPK, ErbB, and AMPK signaling pathways. In addition, the proteins also participated in synapses, mitochondria, ubiquitin–proteasome structures, the autophagy process, and endoplasmic reticulum processes. Next, we speculated that differentially expressed proteins might be involved in the occurrence and development of PND.

## Discussion

Previous studies have shown that open tibial fracture with intramedullary fixation under isoflurane anesthesia impairs the learning and memory ability of adult mice, thereby establishing a PND model [12]. Morris water maze is a common experimental method to evaluate cognitive function in mice [13]. OFT was carried out, as described previously, and the autonomous exercise ability of mice was evaluated by total distance of motion. The hippocampus plays a major role, according to CFT, wherein the fear memory experiment and the sound fear memory experiment are related to the functions of the hippocampus and amygdala [14]. These results showed that compared to the C group, no significant difference was detected in the total distance of exercise in the PND group in the OFT, indicating that the mobility of mice after the operation was not affected. The escape latency was prolonged, the percent residence time in the target quadrant was declined, and freezing time of the contextual fear memory experiment was shortened. These findings indicated that the hippocampus-dependent memory of mice was impaired.

In this study, the mice on days 1, 3, and 7 postoperatively in the PND and control groups were analyzed for differential protein expression. Consequently, 21 proteins in the hippocampus of the PND and control groups differed at the three time points; of these, 12 were upregulated and 9 were downregulated. These proteins participated in different mechanisms and these mechanisms affected the cognitive function of PND mice. The underlying mechanisms may include decreased synaptic plasticity, oxidative stress, and dysregulation of calcium homeostasis caused by mitochondrial dysfunction decreased levels of neuronal autophagy, central nervous inflammatory response, and eventually neuronal loss and apoptosis.

The structural abnormalities of the hippocampal neuronal synapses lead to dementia and cognitive decline [15]. The impaired synaptic plasticity caused by neuronal synapses damage is one of the mechanisms of disordered cognition in PND mice [16]. Stx-1a, belonging to the soluble N-ethylmaleimide-sensitive factor attachment protein receptors (SNARE) protein family [17], is mainly distributed in the cerebral cortex, hippocampus, and amygdala neurons’ presynaptic membrane [18, 19], which is a major component of synapses [20]. Stx-1a regulates calcium-mediated synaptic vesicle exocytosis and transporter activity of neurotransmitters, such as norepinephrine (NA), 5-hydroxytryptamine (5-HT), and dopamine (DA) that affect synaptic plasticity [21]. Previous studies indicated that Stx-1a knockout mice showed long-term impairment of the hippocampus [22], resulting in a significant decrease in the memory and learning ability of mice. Similarly, in the autopsy of patients with advanced AD and dementia, the level of Stx-1a protein in the prefrontal cortex neurons was decreased [23]. Together, these findings suggested that Stx-1a plays a key role in cognitive function. Also, the current demonstrated that Stx-1a protein was upregulated continuously within 7 days after the operation (*P* < 0.05), indicating that synaptic structure and function of PND mouse hippocampus were severely impaired, causing cognitive dysfunction.

Mitochondrial dysfunction has been shown to be a common underlying mechanism of several major neurodegenerative diseases, including Parkinson’s disease (PD), Huntington’s disease, AD, or multiple sclerosis [24–29]. Mitochondrial dysfunction may be involved in cognitive disorder in different ways. Oxidative stress, abnormal energy metabolism, altered calcium ion homeostasis in the endoplasmic reticulum, and endoplasmic reticulum stress caused by mitochondrial dysfunction, were putative mechanisms involved in the decline of cognitive ability [30–32]. Ganglioside-induced differentiation-associated-protein 1 (GDAP-1) is localized in the outer mitochondrial membrane and involved in a variety of physiological processes in mitochondria, including redox and energy production processes and in the regulation of endoplasmic reticulum calcium homeostasis. This proved that GDAP1 improves cognitive function by regulating the level of glutathione, inhibiting the activity of peroxisome in cells, and combating oxidative stress in cells [33, 34]. In addition, neuronal apoptosis mediated by endoplasmic reticulum stress (such as unfolded protein response) may play a role in sevoflurane-induced memory impairment in aging rats [35]. GDAP-1 maintains the mitochondrial–endoplasmic reticulum calcium homeostasis by regulating the store-operated calcium entry (SOCE) process and synaptic plasticity [36], releasing synaptic neurotransmitter normally [37, 38] and restoring the functioning of *N*-methyl-d-aspartic acid (NMDA) receptor for normal learning and memory function [39]. Next, we identified pyruvate dehydrogenase kinase-2 (PDK-2), a kinase that regulates glucose metabolism by closing the pyruvate dehydrogenase complex (PDC) in this experiment [40]. It is also involved in mitochondrial energy metabolism except for GDAP-1. PDK-2 is widely expressed in the mouse hippocampus. Some showed a high level of PDK*-*2 mRNA in the hippocampus of the aged mouse. PDK-2 increases glycolysis and inhibits aerobic metabolism in the mouse hippocampus, which in turn, leads to abnormal energy metabolism and oxidative stress, eventually causing cell damage [41, 42]. The expression of GDAP-1 and PDK-2 was significantly upregulated on days 1, 3, and 7 after the operation, suggesting that PDK-2 may be an injurious factor in the pathological process of PND and affect the energy metabolism of neuronal mitochondria. The upregulation of GDAP-1 indicated its protective role in the pathogenesis of PND.

When exposed to inhaled anesthetics, neuronal cells produce misfolded proteins, such as β-amyloid and α-synuclein [35]. The abnormal aggregation of these misfolded proteins in cells is a critical mechanism of cognitive dysfunction in PND patients [43]. Catechol-O-methyltransferase (COMT) is an enzyme that catalyzes the methylation of catechol substrates: dopamine, estrogen, and polyphenols [44, 45]. The overexpression of COMT promotes α-synuclein and β-amyloid formation in rats. The COMT targets dopamine, elevating the levels of acetylcholine in the brain of AD patients [46]. Moreover, the decrease in dopamine in the brain is also one of the reasons of cognitive disorder in PD patients. COMT also inhibited the processing of β-amyloid precursor protein by estrogen as well as its degradation [45]. Thus, using COMT inhibitors, entacapone and tolcapone, decreased the deposition of α-synuclein and β-amyloid proteins, thereby reducing their induced neurotoxicity and significantly improving the cognitive function of AD and PD patients [47, 48]. The results of the proteomic analysis showed that COMT sustained an elevated expression at the three postoperative time points, hinting at a protective mechanism during PND. Although this mechanism is yet unclear, reducing the expression of COMT, raising dopamine levels in the brain, promoting the degradation of α-synuclein and β-amyloid proteins, and inhibiting their formation could be the putative activities.

Proteasome is the main pathway of misfolded protein degradation during protein biosynthesis [49]. 26S proteasome is composed of 20S core protein and 19S regulatory subunits [50–52]. The decrease in its activity leads to excessive accumulation of misfolded proteins, neurodegenerative symptoms, and neuronal apoptosis in AD and PD patients [53]. Psmc5 protein is involved in the 19S subunit of the proteasome complex. It reduces the level of α-synuclein and β-amyloid protein in cells by recognizing ubiquitinated proteins, repairing misfolded proteins, and transferring misfolded polypeptide to 20S proteasome for hydrolysis [54]. In addition, proteasome is a physiological inhibitor of adenosine 5’-monophosphate (AMP)-activated protein kinase (AMPK) [55]. AMPK participates in apoptosis through downstream target proteins and signaling pathways [56]. Psmc5 is a key junction between 26S proteasome and AMPK that inhibits AMPK via proteasome, thereby preventing cell apoptosis [57, 58]. The expression of Pmsc5 protein in the hippocampus of PND mice was downregulated at the three time points after the operation. Therefore, we speculated that the decreased proteasome activity might be one of the major mechanisms of PND-induced cognitive dysfunction.

Inhaled anesthetics decrease autophagy in the hippocampus of mice, and autophagy is an effective way to remove β-amyloid and α-synuclein proteins from neuronal cells and reduce their neurotoxicity [54]. Furthermore, EGFR is a receptor for epithelial growth factor (EGF) cell proliferation and signal transduction [59, 60] and plays a critical role in cell differentiation during brain development. It is also involved in the differentiation of neural stem cells (NSCs), neural precursor cells (NPCs), and glial precursor cells (GPCs) into neurons and glial cells [61]. The overexpression of EGFR inhibits autophagy through the mTOR pathway. Strikingly, β-amyloid proteins with neurocytotoxicity cannot be degraded, and hence, eventually accumulated in nerve cells. After 3 weeks post-EGFR inhibition by gefitinib and erlotinib, the elimination rate of Aβ-40 and Aβ-42 proteins elevated in amyloid precursor protein/presenilin-1(APP/PS-1) transgenic mice. This phenomenon inhibited the activation of Aβ-42-induced EGFR, reduced the toxicity of Aβ-42 to nerve cells, and improved the learning and cognitive function of APP/PS-1 transgenic mice [62, 63]. Subsequently, the expression of EGFR was upregulated in PND mice, indicating that the function of autophagy was declined and the misfolded proteins were difficult to be eliminated during PND. Some studies have shown that the expression of α-synuclein and β-amyloid proteins in the brain of AD and PD patients increases after general anesthesia, causing further deterioration of cognitive function and other symptoms [64]. In the present study, the downregulation of Psmc5 and the upregulation of EGFR confirms this hypothesis, thereby providing a feasible plan for the treatment of PND.

In a previous study, we found that the concentrations of proinflammatory and anti-inflammatory factors in plasma and the cerebrospinal fluid of PND patients was increased [65], implicating that central nervous inflammatory response is one of the possible pathogenesis mechanisms of PND [66, 67]. In the event of brain tissue and spinal cord injury, the overexpression of EFGR activates the EGFR/MAPK pathway and increases the expression of MAPK, resulting in the transformation of resting astrocytes into reactive astrocytes and secreting proinflammatory cytokines, such as chondroitin sulfate proteoglycans (CSPGs), TNF-α, iNOS, COX-2, and IL-1β. These changes eventually lead to glial scar formation, demyelination, oligodendrocyte formation, and neuronal death [68, 69]. Another protein Rab8a may be involved in inhibiting inflammation in the central nervous system. Rab8a belongs to GTP binding protein, which is involved in the development of neurons, such as axonal growth and dendritic formation [70]. It also recruits PI3K-γ to control the production of cytokines through the mTOR signaling pathway, inhibit the function of NF-κB and the transcription of proinflammatory cytokines IL-6 and IL-12p40, enhance the function of STAT3, and promote the expression of anti-inflammatory cytokine IL-10, in order to reduce the damage of central system inflammation on neurons and improve cognitive function [71, 72]. Additionally, EGFR and Rab8a were significantly upregulated in the hippocampus of PND mice at the three time points after the operation, which confirmed our previous hypothesis that inflammatory response of the central nervous system was a putative mechanism underlying PND [66].

Apoptosis is the final outcome of cell injury [73]. The cognitive function of PND patients was affected by the loss and apoptosis of neurons [74]. Herein, we found that mitochondrial carrier 1 (Mtch1) was continuously upregulated at the three time points after the operation in the PND mice. Mtch1, also known as presenilin-related protein (PSAP), is related to the pathogenesis of AD because it can specifically bind to PS-1 protein. Subsequently, a new apoptotic protein Mtch1 was identified [75], which induces a series of apoptotic processes, such as poly ADP-ribose polymerase (PARP) cleavage, caspase activation, the release of cytochrome C and Smac proteins in mitochondria, eventually leading to neuronal apoptosis [76]. The upregulation of Mtch1 expression in the hippocampus of PND mice indicated apoptosis in the hippocampal neurons, which might be the pathological process underlying the deterioration of cognitive function in PND mice.

Currently, there are only a few proteomics analyses of PND. In this study, high-throughput proteomics techniques were used to screen the proteins that were continuously and differentially expressed on the 1d, 3d, 7d after the operation and verify the various pathological theories of PND. In future studies, we would carry out a follow-up study on differential proteins involving various pathological mechanisms.

Although we use intramedullary needle for internal fixation to reduce the impact of orthopedic surgery on the movement of mice, the behavioral procedures of mice such as the Morris water navigation task will still be affected. At the same time, the lack of further ex vivo/in vitro or randomized controlled trial of the selected proteins in this study is also a limitation. We will continue to verify the specific mechanism of these proteins involved in PND.

## Conclusion

In summary, 21 common differentially expressed proteins were detected on days 1, 3, and 7 after the operation in the hippocampus of PND mice. These proteins are involved in many pathological mechanisms underlying PND, suggesting that the occurrence of PND is the result of multiple mechanisms.

## Data Availability

The datasets used and/or analyzed during the current study are available from the corresponding author on reasonable request.

## Abbreviations

PND: Perioperative neurocognitive disorder
HPLC–MS/MS: High-performance liquid chromatography–mass spectrometry/mass spectrometry
GO: Gene ontology
KEGG: Kyoto Encyclopedia of Genes and Genomes
AD: Alzheimer’s disease
DDA: Data-dependent acquisition
DIA: Data-independent acquisition
DAVID: Database for annotation, visualization, and integrated discovery
MAPK: Mitogen-activated protein kinase
AMPK: Adenosine 5’-monophosphate (AMP)-activated protein kinase
PD: Parkinson’s disease
GDAP-1: Ganglioside-induced differentiation-associated-protein 1
SOCE: Store-operated calcium entry
PDK-2: Pyruvate dehydrogenase kinase-2
COMT: Catechol-O-methyltransferase
EGFR: Epithelial growth factor receptor
Mtch1: Mitochondrial carrier 1
